# Synthesis and Photophysical and Electrochemical Properties of Functionalized Mono-, Bis-, and Trisanthracenyl Bridged Ru(II) Bis(2,2′:6′,2″-terpyridine) Charge Transfer Complexes

**DOI:** 10.1155/2014/570864

**Published:** 2014-04-30

**Authors:** Adewale O. Adeloye, Peter A. Ajibade

**Affiliations:** Department of Chemistry, Faculty of Science and Agriculture, University of Fort Hare, Private Bag X1314, Alice 5700, South Africa

## Abstract

With the aim of developing new molecular devices having long-range electron transfer in artificial systems and as photosensitizers, a series of homoleptic ruthenium(II) bisterpyridine complexes bearing one to three anthracenyl units sandwiched between terpyridine and 2-methyl-2-butenoic acid group are synthesized and characterized. The complexes formulated as bis-4′-(9-monoanthracenyl-10-(2-methyl-2-butenoic acid) terpyridyl) ruthenium(II) bis(hexafluorophosphate) (**RBT1**), bis-4′-(9-dianthracenyl-10-(2-methyl-2-butenoic acid) terpyridyl) ruthenium(II) bis(hexafluorophosphate) (**RBT2**), and bis-4′-(9-trianthracenyl-10-(2-methyl-2-butenoic acid) terpyridyl) ruthenium(II) bis(hexafluorophosphate) (**RBT3**) were characterized by elemental analysis, FT-IR, UV-Vis, photoluminescence, ^1^H and ^13^C NMR spectroscopy, and electrochemical techniques by elemental analysis, FT-IR, UV-Vis, photoluminescence, ^1^H and ^13^C NMR spectroscopy, and electrochemical techniques. The cyclic voltammograms (CVs) of (**RBT1**), (**RBT2**), and (**RBT3**) display reversible one-electron oxidation processes at *E*_1/2_ = 1.13 V, 0.71 V, and 0.99 V, respectively (versus Ag/AgCl). Based on a general linear correlation between increase in the length of *π*-conjugation bond and the molar extinction coefficients, the Ru(II) bisterpyridyl complexes show characteristic broad and intense metal-to-ligand charge transfer (MLCT) band absorption transitions between 480–600 nm, *ε* = 9.45 × 10^3^ M^−1^ cm^−1^, and appreciable photoluminescence spanning the visible region.

## 1. Introduction

Ruthenium(II) polypyridyl complexes have attracted wide attention in the past 30 years because of their use as photosensitizers in the storage of solar energy and in the decomposition of water. This has led to a detailed understanding of their photophysics, photochemistry, and redox chemistry. These complexes have high emission quantum yields, long emission lifetimes, and good redox characteristics. Through a careful choice of ligands and substituents, such complexes have been used in the fabrication of molecular devices, molecular probes, electronics, and sensors [1–10].

In the development of dyes, for example, both absorption in wide range of solar spectrum and high molecular extinction coefficient are required. The black dye exhibits better near-IR photoresponse than the N3 dye, because of its expanded *π* conjugated field. Moreover, the higher efficiencies were obtained with the black dye [11]. On the other hand, a structural modification of the black dye [12–17] is sparse though that of the N3 dye has been widely investigated to increase the stability and the performance. A difficult synthesis of modified terpyridine ligands is one of the major factors. From this point of view, the syntheses of new forms of black dye take an important role in increasing the potential of dyes.

Different authors have prepared several homoleptic and heteroleptic ruthenium complexes using various approaches and, in particular, the extension of the excited-state lifetimes of ruthenium(II) bisterpyridine complexes. The main focus has been on modifications that affect the emitting triplet metal-to-ligand charge transfer (^3^MLCT) states, thus minimizing their interaction with higher-lying ^3^MC states. This has been achieved by the use of substituents [18], cyclometalating ligands [19], and ligands with extended *π* systems [20–22]. Most of these attempts have resulted in a lowering of the ^3^MLCT energy. A few recent studies have more specifically aimed at increasing the ligand field strength by the use of strong *σ*-donor ligands [23, 24] a strategy, however, that results in a concomitant decrease of the ^3^MLCT state energy and, thus, limits the reactivity [25]. The dye solar cells devices based on such complexes, for example, display good photovoltaic performance, and also strong metal-to-ligand charge transition (MLCT) band in the visible region of absorption. Nevertheless, further extension of the conjugation length of the ancillary ligand faces the situation that the *π*-orbital energy of the ancillary ligands increases to match that of the metal centre, with the result that the *π*-orbital of the ancillary ligand participates significantly in the HOMO of the complex. The result is the reduction of the absorption coefficient of the MLCT band and therefore decreased efficiency [26].

This paper reports the recent attempts based on the introduction of anthracene functionality in a stepwise increase into terpyridine ligands and the successful complexation of these ligands to a transition metal ion [ruthenium(II)]. Our research goal is specifically focused on improving the molar extinction coefficient, increasing the wavelength of complexes towards the near-infrared region, and improving dye stability.

## 2. Experimental Section

### 2.1. Materials and General Physical Measurements

All commercial reagents used were analytically pure without further purification. The ligand precursors 4′-(9-bromanthracenyl)-terpyridine (**L**_**1**_), 4′-(9-bromodianthracenyl)-terpyridine (**L**_**2**_), and 4′-(9-bromotrianthracenyl)-terpyridine (**L**_**3**_) were prepared as described in the literature [27]. The thin layer chromatography (tlc) analyses were done with aluminium sheet precoated with normal phase silica gel 60 F_254_ (Merck, 0.20 mm thickness) except otherwise stated. The tlc plates were developed using any of the following solvent systems: Solvent system A: Dichloromethane-Methanol (9 : 1); Solvent system B: Dichloromethane-Methanol (7 : 3); Solvent system C: Dichloromethane-Benzene (3 : 7); Solvent system D: Chloroform-Methanol (1 : 1). Gel filtration was performed using Sephadex LH-20 previously swollen in specified solvent (s) prior to loading of extract onto the column (3.5 cm × 8.5 cm).

Melting points were determined using Gallenkamp electrothermal melting point apparatus. Microanalyses (C, H, N) were carried out with a Fisons elemental analyser and infrared spectra were obtained with KBr discs or nujol on a Perkin Elmer System 2000 FT-IR Spectrophotometer. UV-Vis and fluorescence spectra were recorded in 1 cm path length quartz cell on a Perkin Elmer Lambda 35 spectrophotometer and Perkin Elmer Lambda 45 spectrofluorometer, respectively. ^1^H and ^13^C Nuclear Magnetic Resonance spectra were run on a Bruker EMX 400 MHz spectrometer for ^1^H and 100 MHz for ^13^C. The chemical shift values were reported in parts per million (ppm) relative to (TMS) as internal standard. Chemical shifts were also reported with respect to DMSO d_6_ at *δ*_c_ 40.98 and DMSO d_6_ at *δ*_H_ 2.50 ppm. All electrochemical experiments were performed using Autolab potentiostat PGSTAT 302 (EcoChemie, Utrecht, The Netherlands) driven by the general purpose Electrochemical System data processing software (GPES, software version 4.9). Square wave voltammetric analysis was carried out at a frequency of 10 Hz, amplitude of 50 mV, and step potential of 5 mV. A conventional three-electrode system was used. The working electrode was a bare glassy carbon electrode (GCE); Ag|AgCl wire and platinum wire were used as the pseudoreference and auxiliary electrodes, respectively. The potential response of the Ag|AgCl pseudoreference electrode was less than the Ag|AgCl (3 M KCl) by 0.015 ± 0.003 V. Prior to use, the electrode surface was polished with alumina on a Buehler felt pad and rinsed with excess millipore water. All electrochemical experiments were performed in freshly distilled dry DMF containing TBABF_4_ as supporting electrolyte.

#### 2.1.1. Synthesis of Ruthenium(II)-bis(9-monoanthracenyl-10-(2-methyl-2-butenoic acid)-terpyridyl)-bis(hexafluorophosphate) Complex: **RBT1**

The ruthenium complex precursor, RuCl_2_(DMSO)_4_ and the corresponding ruthenium bisterpyridine complexes were prepared with a slight modification to literature method [28–30]. 4′-(9-bromoanthracenyl)-terpyridine (**L**_**1**_) (0.195 g, 0.40 mmol) was reacted with RuCl_2_(DMSO)_4_ (0.097 g, 0.20 mmol) in DMF (40 mL). The resulting mixture was refluxed for 8 h in the dark and cool to room temperature. Without isolation of the dark red complex, 2-methyl-2-butenoic acid (0.04 g, 0.40 mmol), triethylamine (1 mL), and KOH (0.05 g, 0.80 mmol) were added and the reaction mixture further allowed to reflux for another 4 hours. The crude product was allowed to cool to room temperature and filtered to remove unreacted solid products. Distilled water was added and extracted into chloroform. The organic layer was collected and concentrated to dryness* in vacuo* on rotary evaporator. A 10 mL solution of NaOH (0.05 M) was added and the mixture turned to a deep orange red colour. After filtration, pH was adjusted to 3 with HNO_3_ 0.5 M, and resulting solution was left to stand in the fridge for 12 h. The acidic solution after filtration of the insoluble precipitate was concentrated to afford a semisolid product which was purified by column chromatography on Sephadex LH20 in ethanol-toluene, 50%, v/v). Column fractions with similar tlc characteristics were bulked together and concentrated under reduced pressure and excess ammonium hexafluorophosphate in water was used to precipitate the complex to afford (**RBT1**) as a dark brown solid (0.18 g, Percentage Yield = 32.1%, mp = 196-197°C). IR data (KBr, cm^−1^): 3418, 3121, 2926, 2852, 2366, 1917, 1678, 1621, 1592, 1451, 1384, 1304, 1284, 1256, 1170, 1097, 1054, 1028, 926, 839, 772, 747, 694, 558, 468, 423. *λ*_max⁡_/nm (*ε*/10^3^ M^−1^ cm^−1^) (CH_3_Cl/MeOH, 1 : 1, v/v) 1012 (0.937), 916 (1.074), 486 (5.337), 405 (17.368), 383 (19.391), 364 (15.062). *λ*_em_ = 681 nm (Int. 932%).^ 1^H NMR (400 MHz, DMSO-d_6_): *δ* 9.08 (d, *J* = 8.1 Hz, 2H, H-3′, 5′), 8.81 (d, *J* = 8.0 Hz, 2H, H-3, 3′′), 8.54 (dd, *J* = 3.2, 6.8 Hz, 3H, H-a), 8.21 (dd, *J* = 3.3, 5.8 Hz, 2H, H-b), 8.08 (t, *J* = 7.9, 8.1 Hz, 4H, H-4, 4′′), 7.94 (dd, *J* = 3.2, 5.7 Hz, 1H, H-c), 7.80 (dd, *J* = 3.2, 6.8 Hz, 1H, H-d), 7.42 (d, *J* = 5.3 Hz, 2H, H-6, 6′′), 7.26 (t, *J* = 6.6, 6.8 Hz, 2H, H-5, 5′′), 2.73 (s, CH_3_), 2.56 (s, CH_3_). ^13^C NMR (400 MHz, DMSO-d_6_): *δ* 182.49, 157.69, 154.73, 152.01, 138.06, 135.87, 134.54, 132.99, 130.32, 130.08, 128.45, 127.73, 126.73, 124.49, 123.95, 122.75, 35.75, 34.36. Elemental analysis, Found: C, 58.37; H, 3.65; N, 5.54; Calculated: C, 58.08; H, 3.58; N, 5.98 (Molecular formulae: RuC_68_H_50_N_6_O_4_P_2_F_12_).

#### 2.1.2. Synthesis of Ruthenium(II)-bis(9-dianthracenyl-10-(2-methyl-2-butenoic acid)-terpyridyl)-bis(hexafluorophosphate) Complex: **RBT2**

The complex was prepared using the same procedure as reported for** RBT1**. (**L**_**2**_) (0.25 g, 0.38 mmol) and RuCl_2_(DMSO)_4_ (0.09 g, 0.19 mmol), 2-methyl-2-butenoic acid (0.04, 0.38 mmol), triethylamine (1.0 mL), and KOH (0.05 g, 0.76 mmol). (**RBT2**) was obtained as a deep orange solid; Weight = 0.24 g, Percentage Yield = 45.9%, mp = 195–197°C). IR data (KBr, cm^−1^): 3429, 3076, 2926, 2864, 2272, 1953, 1678, 1616, 1516, 1431, 1384, 1332, 1304, 1285, 1256, 1170, 1053, 1028, 839, 776, 747, 693, 558. *λ*_max⁡_/nm (*ε*/10^3^ M^−1^ cm^−1^) (CH_3_Cl/MeOH, 1 : 1, v/v) 1012 (1.171), 916 (1.343), 554 (2.543), 484 (9.453), 404 (27.783), 383 (28.685), 364 (20.644). *λ*_em_ = 743 nm (Int. 754%). ^1^H NMR (400 MHz, DMSO-d_6_): *δ* 9.08 (d, *J* = 8.2 Hz, 2H, H-3′, 5′), 8.82 (d, *J* = 8.7 Hz, 2H, H-3, 3′′), 8.75 (dd, *J* = 4.7, 8.7 Hz, H-a′), 8.63 (d, *J* = 8.1 Hz, H-b′), 8.60 (dd, *J* = 3.8, 8.8 Hz, H-c′), 8.53 (dd, *J* = 3.4, 6.8 Hz, 3H, H-a′′), 8.21 (dd, *J* = 3.4, 5.8 Hz, 2H, H-b′′), 8.06 (t, *J* = 6.8, 8.0 Hz, 4H, H-4, 4′′), 7.94 (dd, *J* = 3.3, 5.8 Hz, 1H, H-c′′), 7.90 (dd, *J* = 5.0, 5.5 Hz, H-c′), 7.87 (dd, *J* = 6.1, 8.0 Hz, H-d′), 7.79 (dd, *J* = 3.1, 6.8 Hz, 1H, H-d′′), 7.43 (d, *J* = 5.3 Hz, 2H, H-6, 6′′), 7.26 (t, *J* = 6.9, 7.1 Hz, 2H, H-5, 5′′), 2.73 (s, CH_3_), 2.56 (s, CH_3_). ^13^C NMR (400 MHz, DMSO-d_6_): *δ* 182.48, 157.69, 157.09, 156.90, 154.86, 154.74, 152.03, 138.15, 134.53, 132.99, 130.30, 130.07, 129.02, 128.44, 127.72, 127.68, 127.61, 126.72, 124.50, 124.17, 122.76, 122.49, 121.75, 121.19, 35.74, 34.35, 30.74. Elemental analysis, Found: C, 65.29; H, 3.62; N, 4.46; Calculated: C, 65.57; H, 3.78; N, 4.78 (Molecular formulae: RuC_96_H_66_N_6_O_4_P_2_F_12_).

#### 2.1.3. Synthesis of Ruthenium(II)-bis(9-trianthracenyl-10-(2-methyl-2-butenoic acid)-terpyridyl)-bis(hexafluorophosphate) Complex: **RBT3**

The complex was prepared as reported for** RBT1**. (**L**_**3**_) (0.25 g, 0.29 mmol) and RuCl_2_(DMSO)_4_ (0.07 g, 0.15 mmol), 2-methyl-2-butenoic acid (0.03, 0.29 mmol), triethylamine (1.0 mL), and KOH (0.03 g, 0.60 mmol). (**RBT3**) was obtained (Bright orange solid, 0.22 g, Percentage Yield = 36.1%, mp = 205–207°C), IR data (KBr, cm^−1^): 3429, 3075, 3028, 2925, 2852, 1940, 1678, 1621, 1593, 1437, 1385, 1332, 1304, 1285, 1256, 1170, 1027, 926, 839, 746, 694, 578, 558. *λ*_max⁡_/nm (*ε*/10^3^ M^−1^ cm^−1^) (CH_3_Cl/MeOH, 1 : 1, v/v): 1012 (2.217), 916 (1.778), 554 (4.004), 481 (9.130), 402 (45.975), 380 (49.346), 360 (39.677). *λ*_em_ = 734 nm (Int = 754%). ^1^H NMR (400 MHz, DMSO-d_6_): *δ* 9.08 (d, *J* = 8.2 Hz, 2H, H-3′, 5′), 8.82 (d, *J* = 8.7 Hz, 2H, H-3, 3′′), 8.75 (dd, *J* = 4.7, 8.7 Hz, H-a′), 8.63 (d, *J* = 8.1 Hz, H-b′), 8.60 (dd, *J* = 3.8, 8.8 Hz, H-c′), 8.53 (dd, *J* = 3.4, 6.8 Hz, 3H, H-a′′), 8.21 (dd, *J* = 3.4, 5.8 Hz, 2H, H-b′′), 8.06 (t, *J* = 6.8, 8.0 Hz, 4H, H-4, 4′′), 7.94 (dd, *J* = 3.3, 5.8 Hz, 1H, H-c′′), 7.90 (dd, *J* = 5.0, 5.5 Hz, H-c′), 7.87 (dd, *J* = 6.1, 8.0 Hz, H-d′), 7.79 (dd, *J* = 3.1, 6.8 Hz, 1H, H-d′′), 7.43 (d, *J* = 5.3 Hz, 2H, H-6, 6′′), 7.26 (t, *J* = 6.9, 7.1 Hz, 2H, H-5, 5′′), 2.73 (s, CH_3_), 2.56 (s, CH_3_). ^13^C NMR (400 MHz, DMSO-d_6_): *δ* 182.48, 157.69, 157.10, 156.91, 156.82, 154.86, 152.04, 138.20, 134.53, 133.01, 130.31, 128.45, 127.73, 127.62, 126.72, 124.50, 124.17, 123.96, 122.76, 122.49. Elemental analysis, Found: C, 70.33; H, 3.39; N, 3.78; Calculated: C, 70.55; H, 3.92; N, 3.98 (Molecular formulae: RuC_124_H_82_N_6_O_4_P_2_F_12_).

## 3. Results and Discussion

### 3.1. Syntheses

**Scheme 1:**
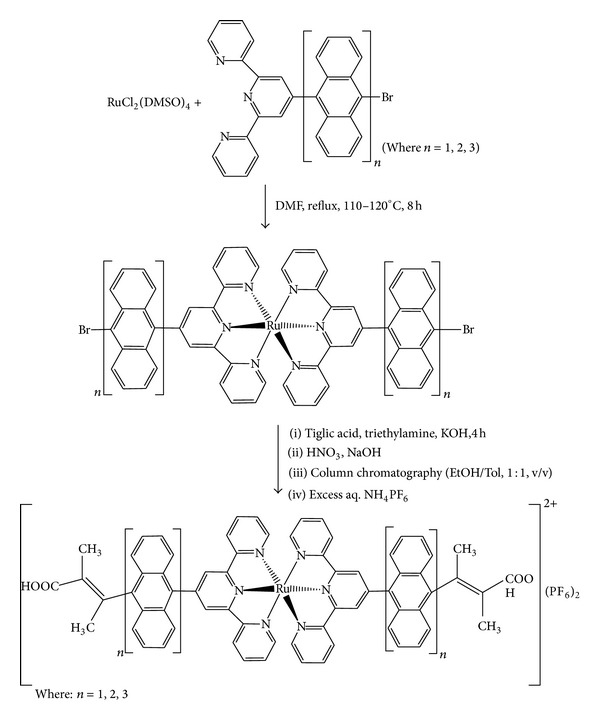
General reaction scheme for preparation of novel Ru(II) bis(terpyridyl-oligo-anthracene) bis(hexafluorophosphate) complexes.

The synthetic route to the formation of the ligands precursors 4′-(9-bromoanthracenyl)-terpyridine (**L**_**1**_), 4′-(9-bromodianthracenyl)-terpyridine (**L**_**2**_), and 4′-(9-bromotrianthracenyl)-terpyridine (**L**_**3**_) has been reported elsewhere [27]. The homoleptic complexes (**RBT1**), (**RBT2**), and (**RBT3**) were prepared following a slight modification of standard procedures [20, 28–30], when two equivalents of the ligand (**L**_**1**_), (**L**_**2**_), or (**L**_**3**_) were reacted with RuCl_2_(DMSO)_4_ in dimethylformamide to form the initial homoleptic bromoanthracenyl terpyridyl ruthenium(II) complexes which were not isolated, and a further subsequent addition of 2-methyl-2-butenoic acid (2 equivalents) in a one-pot synthesis under basic reaction medium (Scheme 1). All complexes were characterized by elemental analyses, FT-IR,^ 1^H, and ^13^C NMR and these were in accordance with the assigned structures.

### 3.2. Infrared Spectra

The infrared spectra of the complexes (**RBT1**), (**RBT2**), and (**RBT3**) share common features, one of which is the strong broad bands in the region of 2737 and 3430 cm^−1^ due to hydroxyl groups of the carboxylic acid moieties on the complexes. The spectral of the complexes display broad asymmetric carboxylate stretching bands *ν*_asym_(COO^−^) in the regions 1950 and 1678 cm^−1^. Both bands are indicative of a protonated carboxylate group on the anthracene. Other common features at the functional group region are the presence of the carboxylate symmetric band at 1384 cm^−1^ *ν*(COO_s_^−^) together with broad *ν*(C=N) of the polypyridyl group at 1593, 1516, and 1451 cm^−1^ [31]. The bands in the regions 1600–1400 cm^−1^ are ascribed to the stretching mode of terpyridine and the alkenyl groups. The presence of broad peaks in the regions 770 and 690 cm^−1^ demonstrates the existence of four adjacent hydrogen atoms which exist in all spectra. These results indicate that the coupling of anthracene rings at the 9, 10-positions led to the formation of polyanthracene chains. The weak absorption frequencies in the region 470 and 420 cm^−1^, respectively, gave indication of the coordination of nitrogen atoms of the ancillary ligands to ruthenium central metal atom [32].

### 3.3. Electronic Absorption and Emission Spectra

#### 3.3.1. Electronic Absorption Spectroscopy

**Figure 1:**
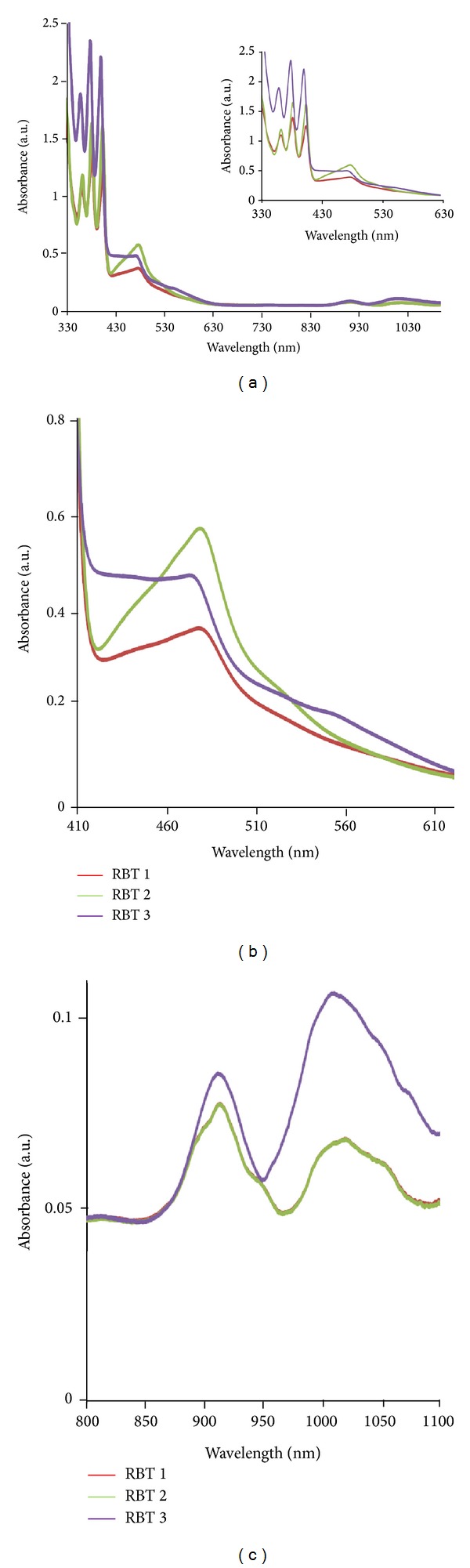
UV-Vis absorption spectra of the (**RBT1**,** RBT2**, and** RBT3**) complexes at a concentration of 0.001 g/dm^−3^ in (CH_3_Cl/MeOH, 1 : 1, v/v).

**Figure 2:**
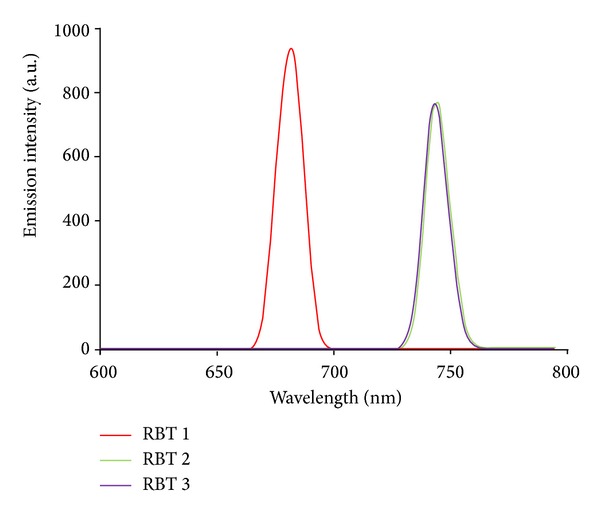
Emission spectra of the** RBT1 **(red),** RBT2 **(green), and** RBT3** (purple) complexes at a concentration of 0.001 g/dm^−3^ in (CH_3_Cl/MeOH, 1 : 1, v/v).

The electronic absorption maxima, molar extinction coefficients, and emission properties of (**RBT1**), (**RBT2**), and (**RBT3**) are listed in Table 1. The absorption, excitation, and emission spectra of the complexes at room temperature in CH_3_Cl/MeOH (1 : 1, v/v) are plotted in Figures 1 and 2, respectively. The three complexes exhibit the typical singlet metal-to-ligand charge transfer (^1^MLCT) absorption bands of ruthenium polypyridyl complexes with maxima at 486, 484, and 481 nm (*ε* = 5.30–9.50 × 10^3^ M^−1^ cm^−1^), respectively. All the complexes showed small but significant shoulder peaks at ~554 nm (*ε* = 1.90–4.00 × 10^3^ M^−1^ cm^−1^) as their extinction coefficients descend steadily towards longer wavelengths. Moreover, there exists weak shoulder bands in the long wavelength tail region of the absorption spectra at 916 nm (*ε* = 1.00–1.80 × 10^3^ M^−1^ cm^−1^) and 1012 nm (*ε* = 0.90–2.20 × 10^3^ M^−1^ cm^−1^); these have been assigned to the triplet metal-to-ligand charge transfer (^3^MLCT) transitions, with complex (**RBT3**) having a molar extinction coefficient better than (**RBT1**) and (**RBT2**) [1, 23, 24]. These bands are similar to that reported for other bipyridyl, phenanthrolyl, and terpyridyl complexes. Groups which extend the delocalization of the *π* systems of polycyclic arenes cause further bathochromic shifts, but the extents of these shifts vary with the positions of substitution [20, 33]. Ligand centered *π* → π* transitions of the terpyridine occur at higher energy in the ultraviolet region between 250 and 300 nm (not shown), while the near-visible region of absorption was characterized by three distinct intense vibronic peaks at 364, 383, and 405 nm for (**RBT1**) and (**RBT2**), and slight blue-shift wavelengths observed for (**RBT3**) at 360, 380, and 402 nm, which may be due to increase in the energy of the LUMO of the ligand. The absorption bands in this region were attributed to wavelength characteristics of anthracene derivatives; however, the molar extinction coefficients of these peaks increase with increase in the number of anthracenes due to extended *π*-bond conjugation in the molecules. The enhanced absorption cross sections between 350 and 405 nm from the anthracenyl chromophores provide an antenna system for the efficient harvesting of UV light (Figure 1). In all the three ruthenium complexes, there are indications that the molecules are strongly coupled and behave more like supermolecules rather than as two individual units. Since the lowest energy anthracene bands are well removed from the [Ru(terpy)_2_]^2+^ transitions, good photoselectivity in this wavelength region can be achieved [18].

#### 3.3.2. Emission Study

Upon excitation into the ^1^LC and ^1^MLCT bands, (*λ*_exc_ = 500 nm), the complexes (**RBT1**), (**RBT2**), and (**RBT3**) display appreciable luminescence at room temperature. However, it was observed that (**RBT1**) showed better emission intensity at lower wavelength (*λ*_em_ = 681 nm) than (**RBT2**) and (**RBT3**) at *λ*_em_ = 743 nm (Figure 2). The intensity of this MLCT transition is unusually high, but strong MLCT bands appear to be a characteristic of “Ru(terpy)” based chromophores bearing a conjugated substituents at the 4′-position [34, 35]. The photophysical properties of “Ru(terpy)” based chromophores are particularly sensitive to the energy gap between emitting MLCT state and a higher energy metal centred state [36]; it is not clear at the moment to adduce the difference in wavelengths between** RBT1**,** RBT2,** and** RBT3** simply to an influenced lowering of the triplet energy. It has been reported that the emission properties of the complexes relate to the substituents on the coordinating nitrogen and their complexity [37]. With the choice of ligands, however, it is well thought that the energy positions of MC, MLCT, and LC excited states of the complexes depend on the ligand field strength [1]. The B3LYP/6-31G theoretical calculations showed that the electronic structures of anthracene derivatives are perturbed by the side substitutes on the anthracene block, and the slight variation of the electronic structures results in the enhanced electron accepting ability and the decrease of the HOMO-LUMO energy gap, which is the origin of the shifting of emission wavelength to the blue-green region [38].

### 3.4. NMR Spectra

The ^1^H NMR spectra of the homoleptic complexes** RBT1**,** RBT2,** and** RBT3** display similarities in the chemical shifts of protons for the terpyridine ligand and the anthracene rings in the aromatic region. The terpyridine protons were conspicuously found as three doublet and two triplet peaks at *δ* 9.08, 8.87, 7.42, and 8.01, 7.26 ppm due to H3′, H5′; H3, H3′′; H4, H4′′; and H6, H6′′; H5, H5′′, respectively. The ^1^H NMR spectra of the complexes also display a characteristic downfield shifts *δ* 8.54, 8.21, 7.94, and 7.80 ppm and AA′BB′ coupling patterns (doublet of doublet) characteristic of* peri* protons (resulting from ^3^ *J* and ^4^ *J*) for the anthracene molecules. Unlike in complex** RBT1**, higher integral values are recorded for these protons in both complexes** RBT2** and** RBT3**, which accounted for the additional anthracene molecules attached to the terpyridine ring. In addition, three prominent singlet peaks at *δ* 7.21, 7.08, and 6.96 ppm were unambiguously assigned to OH peaks in the complexes. In the aliphatic region of the spectra, the singlet peaks at *δ* 2.89, 2.73, and 1.22 ppm were assigned to the methyl protons. The ^13^C-NMR spectra of complexes** RBT1**,** RBT2**, and** RBT3** showed the chemical shifts characteristics of terpyridine and anthracene derivatives. In the aromatic region (*δ* 182–120 ppm), six to eight quaternary carbon resonance signals were observed at 182.49, 157.68, and 156.90, 138.20, 130.31, 123.96, 122.49 ppm, accounting for the carbonyl and the nonhydrogenated carbon atoms in the molecule. The methine carbon resonance peaks of the terpyridyl and anthracenyl molecules are found at 154.86, 152.04, 133.00, 128.45, 127.72, 124.50, and 134.53, 126.72, respectively. The intensity ratio for the methyl carbons at the aliphatic region is too low as compared to the number of anthracenes in the complexes.

### 3.5. Electrochemical Study

**Figure 3:**
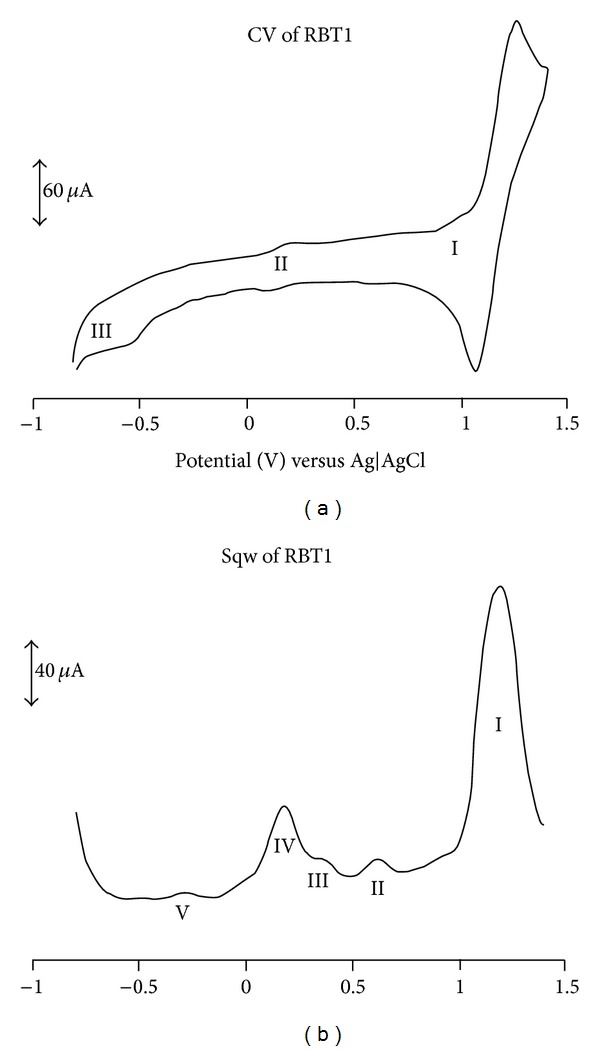
Cyclic and square wave voltammetry profiles of 1 × 10^−3^ M of** RBT1** in freshly distilled DMF containing 0.1 M TBABF_4_ supporting electrolyte. Step potential: 5 mV, amplitude: 50 mV versus Ag|AgCl, frequency: 10 Hz. Scan rate: 100 m Vs^−1^ versus Ag|AgCl.

**Figure 4:**
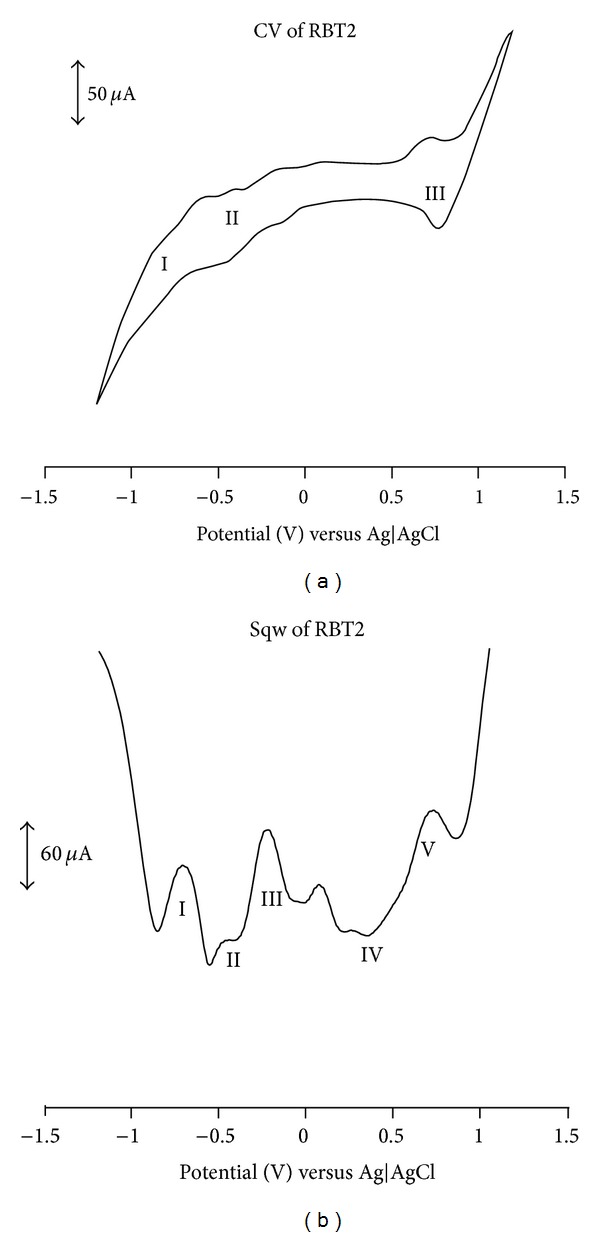
Cyclic and square wave voltammetry profiles of 1 × 10^−3^ M of** RBT2 **complex in freshly distilled DMF containing 0.1 M TBABF_4_ supporting electrolyte. Step potential: 5 mV, amplitude: 50 mV versus Ag|AgCl, and frequency: 10 Hz. Scan rate: 100 m Vs^−1^ versus Ag|AgCl.

**Figure 5:**
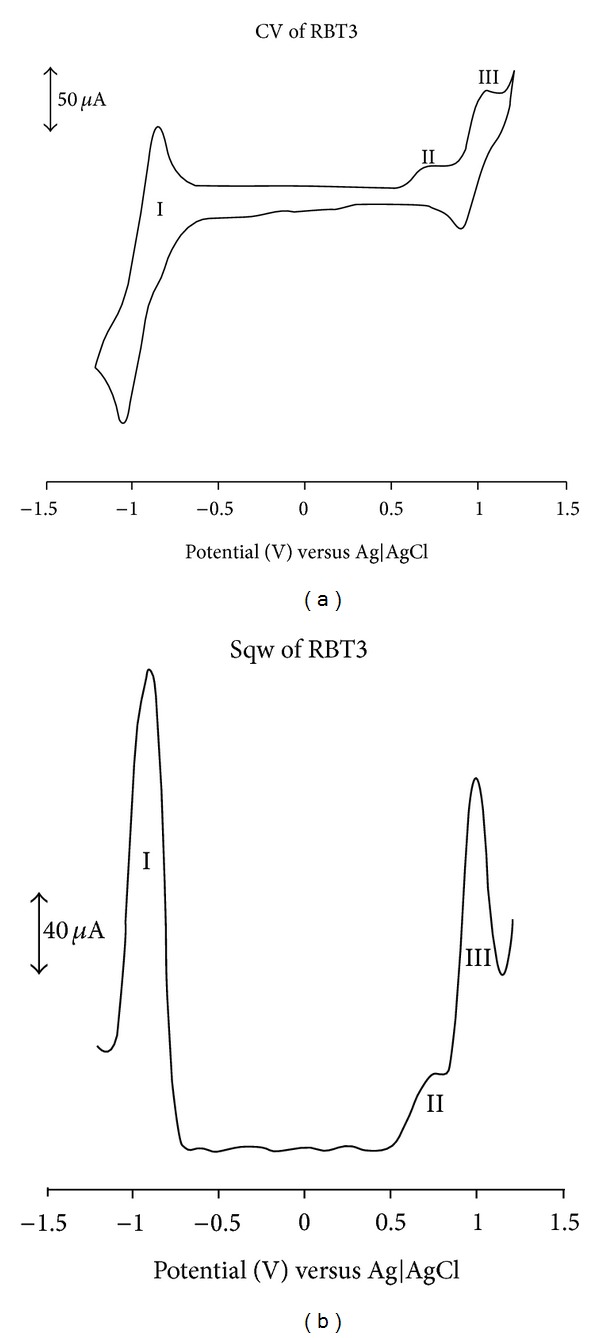
Cyclic and square wave voltammetry profiles of 1 × 10^−3^ M of** RBT3 **complex in freshly distilled DMF containing 0.1 M TBABF_4_ supporting electrolyte. Step potential: 5 mV, amplitude: 50 mV versus Ag|AgCl, frequency: 10 Hz. Scan rate: 100 m Vs^−1^ versus Ag|AgCl.

The cyclic and square wave voltammograms of the (**RBT1**,** RBT2**, and** RBT3**) complexes were examined in the potential range +1.5 to −1.5 V and at a scan rate 50 mV s^−1^ using Ag|AgCl electrode in DMF solvent with 0.1 M tetrabutylammonium hexafluorophosphate as supporting electrolyte. Figures 3, 4, and 5 display the cyclic and square wave voltammogram of** RBT1**,** RBT2**, and** RBT3**, respectively. The voltammograms display the Ru(III)/Ru(II) couple at positive potentials and the ligand-based reduction couples at negative potentials. The potentials are summarized in Table 1. The complexes showed well-defined one-electron oxidation reversible waves at 1.13, 0.71, and 0.99 V for** RBT1**,** RBT2**, and** RBT3**, respectively. These potentials were assigned to the Ru(III)/Ru(II) couple [39]. Other ligand based oxidation potentials for** RBT1** were found at 0.30, 0.57, and 0.85 V. At the negative potential,** RBT1** shows reduction potentials at *E*_1/2_ = −0.25 and −0.54 V. For** RBT2** and** RBT3** complexes, two irreversible oxidation processes** IV** and** II** at 0.28 and 0.73 V, respectively were observed and assigned unequivocally to the ring oxidation of the anthracenyl and/or the terpyridine ligands. At the reduction potential,** RBT2** showed three quasi-reversible reduction peaks processes** I**,** II**, and** III** at *E*_1/2_ = −0.21, −0.46, and −0.69 V. These peaks though were not well defined in the CV but conspicuously shown in the square-wave voltammetry. A reversible reduction peak process** I** at *E*_1/2_ = −0.90 V was observed in** RBT3**. Based on the strong negative potential in** RBT2** and** RBT3** compared to** RBT1**, the influence of conjugation is shown, thus giving the support to the increase in number of anthracene molecular units in the complexes and a corresponding increase in the electron donating ability.

## 4. Conclusions

Terpyridines are versatile and important ligands for a wide variety of transition metal ions. Bis(terpyridyl) ruthenium(II) complexes, in particular, have been used in a diverse range of applications. We have rationally designed and synthesized coordinating Ru(II) bis(terpyridyl) complexes by a stepwise increase in the number of attached anthracene anchored to an *α*, *β*-unsaturated carboxylic acid derivative on terpyridine which have resulted in a better molar extinction coefficient and red shifting of the luminescence towards the near-infrared region. The highly conjugated ruthenium(II) terpyridyl complexes (**RBT1**,** RBT2**, and** RBT3**) combine the organic semiconductor property of anthracene and extended *π*-conjugation of the acid functionality, thus leading to an enhanced molar extinction coefficients. The results obtained in this study gave indication that the luminescence property such as the lifetime of complexes is possible through synthetic control of the number of anthracenyl group units, since prolongation of MLCT lifetimes is valuable for the advancement of analytical luminescence-based technologies such as lifetime-based sensing [40–44]. However, an optimum number of anthracenes may be required for a better electron transfer processes as well as reduction in molecular aggregation. The complexes investigated showed better photophysical and electrochemical characteristics which may endeared them to be used as suitable candidates for larger supramolecular systems based on Ru(II) polypyridine compounds capable of performing long-range photoinduced electron and/or energy transfer functions, photosensitizers, electronic devices, and components in molecular assemblies for generating charge-separated states. To support the potential capability of these complexes as sensitizers for dye-sensitized solar cells, the photovoltaic and electrochemical impedance spectroscopy experiments are in progress.

## Figures and Tables

**Table 1:** Photophysical properties and electrochemical data of **RBT1**, **RBT2,** and **RBT3** complexes.

Complex	*λ*_abs_/nm (*ε* × 10^3^/M^−1^ cm^−1^)^a^					*λ*_em_/(nm)^b^	*E*_pa_/V^c^	*E*_1/2_/V^c^
**RBT1**	364 (15.1)	383 (19.4)	405 (17.4)	486 (5.3)	554 (1.9), 916 (1.1), 1012 (0.9)	681 (932)^i^	0.30, 0.57, 0.85, 1.13	−0.25, −0.54

**RBT2**	364 (20.6)	383 (28.7)	404 (27.8)	484 (9.5)	554 (2.5), 916 (1.3), 1012 (1.2)	743 (754)^i^	0.28, 0.71	−0.21, −0.46, −0.69

**RBT3**	360 (39.7)	380 (49.4)	402 (45.9)	481 (9.1)	554 (4.0), 916 (1.8), 1012 (2.2)	743 (754)^i^	0.73, 0.99	−0.90

^a,b^(CH_3_Cl/MeOH, 1 : 1, v/v), ^c^freshly distilled DMF containing 0.1 M TBABF_4_ supporting electrolyte, ^i^emission intensity.
